# P099 More than meets the lung: MALT lymphoma presenting with pulmonary nodules and consolidation in primary Sjögren’s syndrome

**DOI:** 10.1093/rap/rkag113.129

**Published:** 2026-11-03

**Authors:** Srivindiya Ramanaidu, Arabella Waller

**Affiliations:** Maidstone and Tunbridge Wells NHS Trust, Maidstone, United Kingdom; Maidstone and Tunbridge Wells NHS Trust, Maidstone, United Kingdom

## Abstract

**Introduction:**

A 36-year-old man presented to the medical team with fever, cough, arthralgia, lethargy, drenching night sweats and exertional dyspnoea. His medical history included longstanding Sjögren’s syndrome, diagnosed at eight years of age following bilateral parotid swelling and positive SSA/SSB antibodies, seropositive rheumatoid arthritis and a vasculitic ankle ulcer. Blood investigations demonstrated an elevated C-reactive protein with a normal white cell differential. Chest X-ray revealed increased right upper zone opacity, considered most likely infective in the context of immunosuppression. Oral antibiotics were initiated; however, persistent symptoms prompted urgent outpatient computed tomography of the chest, abdomen and pelvis.

**Case description:**

His medication history was notable for previous tapering courses of prednisolone, oral methotrexate, which had resulted in resolution of a prior vasculitic ulcer, and a single course of rituximab administered as treatment escalation for inflammatory arthritis and vasculitic leg ulceration. An urgent CT scan of the chest, abdomen and pelvis demonstrated multiple areas of consolidation and nodularity within the right hemithorax. In view of these findings, he was referred to the lung multidisciplinary team (MDT). Following MDT discussion, navigational bronchoscopy targeting lesions within the right upper and middle lobes was performed; however, the results were non-diagnostic. Subsequent positron emission tomography-computed tomography (PET-CT) demonstrated moderately FDG-avid irregular pulmonary nodules within the right apex and middle lobe, with no evidence of large-vessel vasculitis elsewhere.

The patient was reviewed by the respiratory team and underwent CT-guided biopsy, despite a recognised increased risk of bleeding due to the surrounding cystic component of the lesion. Histological analysis demonstrated benign tissue fragments only. Given his persistent symptoms, including ongoing cough and exertional dyspnoea, a repeat CT-guided biopsy was performed but again showed no evidence of malignant cells. Owing to the persistent radiological abnormalities, progressive symptoms and continued diagnostic uncertainty, he was referred to the thoracic surgery team for consideration of video-assisted thoracoscopic surgery (VATS).

He subsequently underwent VATS wedge resections of the right upper and middle lobes. Histopathological examination confirmed extranodal marginal zone lymphoma of mucosa-associated lymphoid tissue (MALT) type with associated non-amyloid deposition. He was reviewed by the haematology team and commenced treatment with bendamustine and rituximab. His clinical course was complicated by fever, malaise, diarrhoea and a widespread maculopapular rash requiring hospital admission for treatment with intravenous corticosteroids and antihistamines. He was subsequently referred to the allergy and immunology service with suspected rituximab hypersensitivity and is currently awaiting repeat skin testing for further evaluation.

**Discussion:**

Patients with Sjögren’s syndrome have a substantially increased risk of developing non-Hodgkin lymphoma, with extranodal marginal zone lymphoma of mucosa-associated lymphoid tissue (MALT) accounting for approximately 65% of cases. The pathogenesis is thought to involve chronic antigenic stimulation, persistent B-cell activation, impaired immune surveillance and acquired genetic alterations. In this patient, constitutional symptoms, including malaise and drenching night sweats, together with persistent cough and exertional dyspnoea despite appropriate antibiotic therapy, raised suspicion for an underlying lymphoproliferative disorder.

MALT lymphoma most commonly involves the parotid glands in patients with Sjögren’s syndrome; however, extranodal disease affecting the thyroid, lung and other mucosal sites has also been reported. Clinically significant pulmonary involvement occurs in approximately 10–20% of patients with Sjögren’s syndrome, although pulmonary MALT lymphoma remains uncommon. Characteristic radiological features include solitary or multiple pulmonary nodules, areas of consolidation and cystic change. In the present case, the differential diagnosis included pulmonary lymphoma, lymphocytic interstitial pneumonia, infection, pulmonary amyloidosis and organising pneumonia, all recognised pulmonary manifestations of Sjögren’s syndrome.

FDG PET-CT is a valuable adjunct in the diagnostic evaluation of suspected pulmonary lymphoma, facilitating identification of metabolically active lesions suitable for biopsy while excluding alternative diagnoses, including large-vessel vasculitis. Although studies have demonstrated that most biopsied pulmonary nodules in patients with primary Sjögren’s syndrome are malignant, with lymphoma representing the commonest diagnosis, tissue diagnosis may remain challenging. Despite navigational bronchoscopy and two CT-guided biopsies, histological confirmation was not achieved in this case. Definitive diagnosis required video-assisted thoracoscopic surgery (VATS) with wedge resection, highlighting the importance of pursuing surgical biopsy when clinical suspicion remains high despite repeated non-diagnostic minimally invasive investigations. Early recognition, multidisciplinary collaboration and timely histological confirmation are essential to facilitate appropriate treatment and optimise clinical outcomes.

**Key learning points:**

• This case underscores the importance of maintaining a high index of suspicion for lymphoproliferative disorders in patients with Sjögren’s syndrome, particularly when presenting with constitutional symptoms such as malaise and night sweats, alongside persistent respiratory symptoms including cough and dyspnoea. Although the patient was initially treated empirically for presumed pneumonia in the context of immunosuppression, the lack of clinical improvement prompted escalation of investigations. Subsequent CT imaging and acute medical review were crucial in identifying an alternative pathology and facilitating further diagnostic assessment.

• A key learning point from this case is the importance of avoiding diagnostic anchoring, whereby new pulmonary abnormalities in patients with Sjögren’s syndrome are attributed solely to autoimmune-related lung disease or infection. While interstitial lung disease, recurrent infection and treatment-related complications are recognised in this population, persistent or atypical radiological findings should prompt consideration of malignancy, including pulmonary MALT lymphoma, particularly in the presence of systemic symptoms.

• This case also highlights the importance of a multidisciplinary approach in managing diagnostic challenges. Collaboration between the lung multidisciplinary team, radiologists, respiratory physicians and thoracic surgeons was essential in guiding investigations, particularly in the setting of multiple non-diagnostic procedures. This coordinated approach enabled definitive tissue diagnosis through surgical biopsy, allowing haematological treatment to be initiated.

• Furthermore, this case emphasises the need for research into pulmonary involvement and MALT lymphomagenesis in Sjögren’s syndrome, given the rarity and diagnostic complexity of this presentation. Pulmonary manifestations often present with non-specific symptoms, including cough, dyspnoea and fatigue, which may overlap with benign pulmonary complications of Sjögren’s syndrome or treatment-related adverse effects, contributing to delayed diagnosis. There remains limited consensus regarding optimal imaging modalities, biomarkers and screening strategies for detection of pulmonary lymphoma. Addressing these gaps will be essential to improve recognition, outcomes and personalised management for patients at risk of pulmonary lymphoproliferative disease.

